# Mucoadhesive nanoformulations and their potential for combating COVID-19

**DOI:** 10.2217/nnm-2021-0287

**Published:** 2021-11-03

**Authors:** Parteek Prasher, Mousmee Sharma

**Affiliations:** ^1^UGC Sponsored Centre for Advanced Studies, Department of Chemistry, Guru Nanak Dev University, Amritsar, 143005, India; ^2^Department of Chemistry, University of Petroleum & Energy Studies, Energy Acres, Dehradun, 248007, India; ^3^Department of Chemistry, Uttaranchal University, Arcadia Grant, Dehradun, 248007, India

**Keywords:** acute respiratory distress syndrome, cytokine storm, human angiotensin-converting enzyme II, mucoadhesive, mucus hypersecretion, nasal sprays, pulmonary delivery, repurposed drug, SARS-CoV-2, spike glycoprotein

## COVID-19 & mucus hypersecretion

The cytokine storm associated with the pathophysiology of COVID-19 (SARS-CoV-2) arises from an aggressive inflammatory response by the hyperactive immune system in response to the virus invasion, characterized by excessive production of the proinflammatory cytokines, mainly IL-6 and TNF-α. These events result in mucus cell metaplasia marked by mucus hypersecretion that apparently obstructs the respiratory airways, thereby manifesting recurrent airway infections [1]. Computerized tomography (CT) images of the lungs of COVID-19 patients demonstrated the occurrence of mucoid impaction [2], while increased mucous viscosity and sputum volume further confirm the role of mucus hypersecretion in COVID-19 pathogenesis. In addition, the incidence of excessive pulmonary mucous secretion, mucus plugging and the appearance of fibrinous exudates in the alveoli as revealed by the postmortem study of the lungs of confirmed COVID-19 patients uphold the concomitant role of mucus hypersecretion in COVID-19 related complications [3]. The autopsy report on lung tissue samples of 38 COVID-19 patients in a multicenter study performed at the peak of the first wave in worst-hit Northern Italy indicated the presence of dense mucoid material within the lumen of bronchi and bronchiolar branches [4]. The postmortem COVID-19 histopathological examinations conducted in FL, USA on an old-aged couple and a middle-aged person with severe COVID-19 symptoms revealed the incidence of copious mucinous secretions along the lower respiratory tract [5]. Furthermore, a striking 33% of the COVID-19 autopsies conducted at the University Hospital Basel and at the Cantonal Hospital Baselland, Switzerland indicated tracheobronchitis or severe mucoid tracheitis alongside the pathological changes in the distal respiratory tract [6]. The mucus hypersecretion coupled with fibrinous and serous exudation further impair the airway gas-exchange mechanism, thereby causing hypoxemia. The absence of an immediate therapeutic intervention to counter the mucus hypersecretion and mucoid impaction eventually results in the development of acute respiratory distress syndrome (ARDS) that causes acute lung injury followed by multiple organ failure.

## Exigencies in the pulmonary drug delivery during COVID-19

Mucus secretion is an obligatory physiological mechanism that provides the first-line defense to the underlying airway epithelia against invading pathogens. However, the overproduction of mucus during respiratory ailments, including COVID-19, further exacerbates the disease pathogenesis. Mucus hypersecretion also impedes pulmonary delivery of therapeutics, as they fail to penetrate the thick mucus wall overlaying the target morbid epithelia. The administration of pharmaceuticals aimed at managing respiratory disorders benefits from the pulmonary delivery route owing to the large surface area for rapid absorption into systemic circulation and for achieving prompt local action at the target area [7]. However, the presence of copious amounts of mucus discourages the pulmonary route, which encumbers the optimal therapeutic performance of the administered pharmaceutical. This necessitates the development of mucus-penetrating and mucoadhesive drug delivery systems for improving the epithelial contact, drug absorption and its residence time in the airway epithelia, in addition to decreasing the mucociliary clearance rate [8]. Considering the high surface area for absorption into systemic circulation, administration through the oral route presents another feasible approach. However, drugs administered via this route must achieve an optimal bioavailability in the GI tract and prevent their degradation by the acidic pH in the upper gastrointestinal (GI) tract and hydrolyzing enzymes [9]. Therefore, nasal administration of COVID-19 therapeutics in the presence of mucoadhesive or mucus-penetrating particles proves highly advantageous for realizing a prompt drug response, owing to its improved absorption across the airway mucosa.

## Advantages of nanosize in pulmonary delivery

Nanoparticles readily penetrate the mucus mesh comprising a heterogeneous spacing ranging from 20–1800 nm across different organs and disease conditions, where the size-filtering and interaction-filtering mechanisms govern its permeability. Nanoformulations permit the delivery of poorly water-soluble drugs across the pulmonary route, thereby improving drug bioavailability. The nanoparticles incorporated in the mucoadhesive polymeric formulations promote drug localization in the nasal cavity [10]. The use of mucoadhesive natural polymers as excipients along with the aerosolized drug nanoformulation prolong the retention of the latter in the respiratory tract after inhalation. Therapeutic nanoformulations designed for pulmonary delivery, when coated with mucoadhesive nonionic polymers, led to an improved pharmacokinetic profile of the former, as compared with their noncoated counterparts. Similarly, the encapsidation of immunogens in the mucoadhesive nanopolymers provided a sustainable strategy for the intranasal delivery of vaccines aimed at capping viral infections such as COVID-19 [11].

## Mucoadhesive polysaccharide nanoparticles as prospective delivery systems for COVID-19

Mucoadhesive and mucus-penetrating nanoformulations of polysaccharides present a robust candidature for overcoming the contemporary limitations faced during the utilization of the pulmonary drug-delivery route. Pulmonary administration ensures rapid absorption of the administered drug due to high vascularization of the respiratory epithelia, in addition to bypassing first-pass metabolism. Nanosized, neutral drug-delivery vehicles efficiently penetrate through the airway mucus, whereas the delivery systems with a positive surface charge preferentially adhere to the negatively charged mucus mesh in a typical pulmonary delivery paradigm [10]. Importantly, the mucoadhesive delivery systems based on cationic polysaccharides, such as trimethyl chitosan, cationic cellulose and cationic starch, display a prolonged retention time in the airway epithelium, thereby ensuring optimal drug absorption at the target site. The application of nebulizers for the pulmonary delivery of drug-loaded mucoadhesive vehicles provides a noninvasive and portable mode of administration with higher efficacy owing to optimal retention of the drug molecules at the target site. Novochizol represents a first-in-class advanced drug-delivery nanosystem for COVID-19 therapeutics that comprises mucoadhesive cationic chitosan nanoparticles whose aerosol formulation strongly adheres to the mucus layer overlaying the lung epithelia. This results in the realization of sustained drug release and optimal drug absorption at the target site while avoiding the undesirable systemic distribution of the drug [12]. The chemical modification of chitosan to obtain positively charged N,N,N-trimethylchitosan (TMC) alleviates the water solubility of native chitosan at the physiological pH of 7.4, in addition to improving the contact time and retention of the polysaccharide with the mucus layer. These properties of cationic TMC enhance the absorption of peptides and large hydrophilic drug molecules across the mucosal layer at neutral and basic pH, at which the unmodified chitosan becomes ineffective [13]. The recently developed diaminated cationic starch presented a tenfold increase in mucoadhesive potency compared with chitosan due to the protonation of both -NH_2_ and -NH- groups of the former at acidic pH, compared with the protonation of only -NH_2_ group of chitosan at the same pH. Monoaminated cationic cellulose and starch present substantial mucoadhesive profiles at pH below 6.5 [14]. Notably, the cationic nature of the polysaccharide enables electrostatic interactions with the sialic acid component of mucus, hence offering mucoadhesive characteristics. Owing to these remarkable characteristics, mucoadhesive polysaccharide nanoparticles with a surface positive charge are potential drug-delivery systems for COVID-19 therapy.

## Mucoadhesive nanocomposites as prospective oral delivery systems for COVID-19

Yu *et al.* intercalated the COVID-19-repurposed anthelminthic drug niclosamide with 2D nanoclay material (montmorillonite; MMT) lattice via ion–dipole interaction, and its further coating with nonionic polymer surfactant Tween 80 improved the oral bioavailability and aqueous solubility of the drug. The reported hybrid nanosystem improved the release rate of the drug compared with the nonintercalated niclosamide, in addition to ameliorating bioavailability under GI settings. The mucoadhesive property of MMT arises due to the interaction of its -OH groups with the mucous layer via strong hydrogen bonding interactions, which improves the drug pharmacokinetics as compared with nonintercalated drugs by enhancing the concentration of the drug in plasma and ameliorating its retention time in systemic circulation [15]. Niclosamide-loaded hydrotalcite-composite nanohybrids reported by Choi *et al.* with particle sizes <300 nm, further coated with hydroxypropyl methylcellulose (HPMC) or Tween 60 presented an excellent profile for the oral administration of the drug. The oral administration of the reported composite nanohybrids in animal models led to the retention of the optimal therapeutic concentration of the drug in plasma for the treatment of COVID-19. The mucoadhesive properties of hydrotalcite, Tween 60 and HPMC also ameliorated the pharmacokinetic profile of niclosamide by providing better retention and absorption across the mucosal layer. Importantly, a single administration of the reported composite nanohybrids maintained the therapeutic concentration of niclosamide much above its IC_50_ value in 8–12 h, ensuring a long-lasting effect of the cargo drug molecules [16].

## Mucosal delivery of COVID-19 vaccines via the nasal route

The mucosal immune response represents the first-line defense against the invading SARS-CoV-2 infection. Apparently, the respiratory tract serves as the natural route of SARS-CoV-2 infection, while the contemporary SARS-COV-2 vaccines approved for humans display efficacy when administered mainly via the intramuscular route. Mucosal vaccine administration via the nasal route proves advantageous as it enables sterilization and induces localized immunity to the mucosal lining. However, intranasal vaccines face challenges such as physical barriers and the nature of viral antigens. The presence of a suitable adjuvant possessing immunomodulating properties promotes suitable candidates as vaccine-delivery systems. As such, the application of mucoadhesive drug delivery vehicles for the intranasal delivery of COVID-19 vaccines such as SINO-Vac and BB154 stimulates a broad immune response and neutralizes mucosal IgA and IgG, further validating the use of mucus-adhering delivery systems for COVID-19. Here, the presence of mucoadhesive materials serves as an auxiliary agent to enhance the efficacy of the administered vaccines [11]. Jearanaiwitayakul *et al.* optimized the immunogenicity of the receptor-binding domain (RBD) of the SARS-CoV-2 spike glycoprotein impregnated on mucoadhesive TMC nanoparticles. The *in vivo* analysis indicated that the intranasal administration of RBD-TMC nanoparticles induced a notable local mucosal immunity as evidenced by the upregulation of IgG and IgA in the bronchoalveolar lavage fluid. Importantly, the test animals demonstrated a marked antibody response including serum IgA, IgG, IgG1 and IgG2a, in addition to neutralizing antibodies. Furthermore, the immunized mice displayed higher levels of activated splenic CD4^+^ and CD8^+^ cells compared with animals administered soluble RBD immunogen [17]. The intranasal delivery of RBD-TMC nanoparticles triggered a local immune response, in addition to stimulating systemic immunity. These investigations provided an alternate route for vaccine administration that mimics the natural route of SARS-CoV-2 infections.

## Inhalable hACE2-containing nanocatchers for reducing SARS-CoV-2 transmission via nasal administration

There have been successive COVID-19 waves mainly due to the increased number of mutated strains of SARS-CoV-2. The mutations occurring on the spike glycoproteins ameliorate the binding affinity of the virus to the receptors of hACE2. These mutations led to a higher transmission of SARS-CoV-2 and reduced the efficacy of vaccines in countering the mutated strains. Zhang *et al.* designed hACE2-containing nanocatchers that compete with the host cell in binding to the virus, thereby protecting the cells from the SARS-CoV-2 infection. These nanocatchers derived from the cell membranes of genetically engineered cells expressed hACE2 and demonstrated considerable neutralization potency against the pseudoviruses of wild-type SARS-CoV-2 and D614G variants. Further mixing of hACE2-containing nanocatchers with the mucoadhesive excipient hyaluronic acid yielded an inhalable formulation that considerably prolonged the retention of the nanocatchers in the lungs postinhalation. The inhalation of the reported nanoformulation resulted in significant inhibition of the pseudovirus in hACE2-expressing animal models while producing trivial side effects. Importantly, the inhalable hACE2-containing nanocarriers allow long-term storage in a lyophilized formulation [18].

## Nanoformulated nasal sprays for combating COVID-19 transmission

Nasal sprays form a cornerstone therapy for the prevention of virus transmission. Carrageenan demonstrates wider, nonpharmacological antiviral properties and translational advantages that make it an ideal candidate for antiviral nasal sprays. However, poor mucoadhesion limits the advantages of carrageenan in the development of nasal sprays. The supplementation of carrageenan with mucoadhesive excipients such as gellan overcomes these limitations [19]. The nasal sprays based on a composite mixture of gellan and λ-carrageenan proved highly efficacious in preventing SARS-COV-2 infection in Vero cells. The inhibition of the infection occurs via the formation of a steric barrier at the interface of the host cell, followed by the adsorption of polymer to the virus leading to the physical entrapment of the virus in the sprayed layers. The charge–charge interactions between the host cell and virus membrane mainly expedite the adsorption of the polymer [20]. The development of carrageenan-gellan nanoformulations holds promising potency in the development of nasal sprays for combating COVID-19 transmission.

## Conclusion

Mucus hypersecretion represents a characteristic feature associated with the COVID-19 cytokine storm that impairs drug delivery to the affected tissues via the pulmonary route. Nanoformulations prove to be a stalwart tool for achieving the delivery of the pharmaceutical across the airways. Mucoadhesive and mucus-penetrating nanoformulations promote pulmonary drug delivery by evading the mucus barrier while maintaining an optimal drug concentration across the epithelia and rapid absorption into systemic circulation. Mucoadhesive polymers present applications as excipients along with the drug molecules to provide enhanced mucus penetration and a superior pharmacokinetic profile. Tethering of mucoadhesive polymeric particles with the RBD of the SARS-CoV-2 spike glycoprotein enabled the intranasal delivery of the nanoformulation and triggered the local immune response while boosting the systemic immune response. hACE2-containing nanocatchers with the mucoadhesive excipient ‘hyaluronic acid’ provided an inhalable formulation that effectively inhibited the pseudoviruses of wild-type SARS-CoV-2. Hence, nanoformulations with mucoadhesive properties present attractive options for capping the emerging strains of SARS-COV-2 to prevent further emergence and spreading of successive waves of COVID-19.
